# *Vibrio cholerae* was found in cultured bullfrog

**DOI:** 10.1017/S0950268822000164

**Published:** 2022-02-08

**Authors:** Yibin Yang, Xia Zhu, Haixin Zhang, Yuhua Chen, Yongtao Liu, Yi Song, Xiaohui Ai

**Affiliations:** 1 Yangtze River Fisheries Research Institute, Chinese Academy of Fishery Sciences, Wuhan 430223, China; 2 The Key Laboratory for Quality and Safety Control of Aquatic Products, Ministry of Agriculture, Beijing 100037, China; 3 Jiangxi Fisheries Research Institute, Nanchang, China; 4 Department of Gastroenterology, Zhongnan Hospital of Wuhan University, Wuhan 430227, China; 5 Hubei Clinical Center & Key Lab of Intestinal & Colorectal Diseases, Wuhan 430227, China

**Keywords:** Bullfrog, fatal threat, food safety, route of transmission, *Vibrio cholerae*

## Abstract

Bullfrog is one of the most important economic aquatic animals in China that is widely cultured in southern China and is a key breed recommended as an industry of poverty alleviation in China. During recent years, a fatal bacterial disease has often been found in cultured bullfrogs. The clinical manifestations of the diseased bullfrogs were severe intestinal inflammation and an anal prolapse. A bacterial pathogen was isolated from the diseased bullfrog intestines. The bacterium was identified as *Vibrio cholerae* using morphological, biochemical and 16S rRNA phylogenetic analysis. In this study, *V. cholerae* was isolated and identified in diseased bullfrogs for the first time, providing a basis for the diagnosis and control of the disease. Therefore, attention should be paid to the modes of transmission of *V. cholerae* from bullfrog and formulate reasonable safety measures.

## Introduction

Bullfrog belongs to the Ranae family and Rana genus in the order Anura and class Amphibia of the phylum Chordata. It is the most popular large edible frog in the world and is named based on the loud sound it makes and its resemblance to the noise made by cattle [1]. The original distribution of the Bullfrog is to the east of the Rocky Mountains in the United States, in 30° to 40° north latitude area and southern Ontario and Quebec areas in Canada. It is the largest frog found in North America [2] and the adult frog is generally 8–12 cm long and can reach a maximum weight of 2 kg. Although bullfrogs are about 300 million years old, its artificial culture spans only about a hundred years. Due to the nature of its skin, oil, hormones, glands and bile extracted are of economic value, and are used as important raw materials in aquaculture, medicine and other industries [1]. Therefore, bullfrog has been favoured by many consumers since it was first introduced into China from Cuba in 1959 [3]. It is one of the main economically valuable aquaculture animals in China. At present, bullfrog breeding is mainly distributed in Guangdong, Fujian, Zhejiang, Jiangxi, Hainan, Anhui, Jiangsu, Hunan, Hubei, Sichuan and other southern regions [1].

During the raring of bullfrogs, farmers use various techniques to improve yield, and little attention has been paid to the carrying capacity of the waterbodies, causing an increase in the seriousness of diseases along with an increase in breeding density. The continuously increasing scale and density of bullfrog breeding has resulted in many problems, such as the shortage of biologically healthy food, the degradation of germplasm resources, the deterioration of the breeding environment and a lack of breeding technology. The number of diseases encountered during bullfrog breeding is on the rise and are becoming increasingly more detrimental, with frequent large-scale outbreaks that have seriously hindered the development of the industry. At present, the main disease pathogens of bullfrog are bacteria, viruses and parasites [3–5]. Due to the characteristics of many types of pathogens, complex and diverse causes, rapid spread and high mortality rates, bacterial diseases are the most harmful to the bullfrog breeding industry, and have become the focus of prevention and control during the process of bullfrog breeding [5].

During recent years, a strange disease has often broken out in bullfrog farms in the Zhangzhou area of Fujian Province, which is commonly known as anorectal disease by local farmers. The main clinical symptoms of the diseased bullfrogs are anal abscission, rotten faeces and signs of severe dyspepsia, which have been confirmed as severe enteritis through diagnosis. To find out the cause of the disease in bullfrogs as soon as possible, and to formulate prevention and control measures, the aetiology of the disease was studied in bullfrogs with typical symptoms. Bacteriological studies were conducted and several dominant strains were isolated from different batches of samples. Further studies found the same dominant bacteria in most samples based on morphology and dominance.

## Materials and methods

### Sampling

Bullfrogs with typical symptoms were collected multiple times from Zhangzhou, Fujian Province. Diseased bullfrogs with typical symptoms were collected in net bags and brought back to the laboratory for diagnosis and pathogen isolation. Bullfrogs (50 ± 2 g) were purchased for infection experiments from farms without a history of disease. The purchased bullfrogs showed a strong jumping ability and had no scars on their bodies. The healthy bullfrogs were kept in buckets for 7 days, and the water used for breeding was not higher than the neck of the frogs. Seven days later, the bullfrogs were used in infection tests if they were without disease symptoms. All experimental bullfrogs were anesthetised before tissue removal and euthanised after tissue removal.

### Pathogen isolation

A light microscope was used to observe the intestinal tract to identify typical symptoms of parasitic or fungi infections. Bacterial isolation was performed in a secondary biosafety cabinet (ESCO, Singapore). The anesthetised bullfrogs were placed on ice and disinfected using 75% ethanol before dissection. After the intestinal and visceral tissues of each bullfrog were allowed contact with the inoculation ring, the inoculation ring was placed on an agar plate with brain heart extract (BHI; Difco, USA), and the plate was cultured at 28°C for 24 h. The dominant strain was selected and then purified. The purified strain was temporarily named as NW01. The purified strain was mixed with 15% glycerol and frozen at −80°C until use.

### Biochemical characterisation of the bacterial isolates

The NW01 isolated was inoculated into agar medium plates with brain heart extract and cultured at 28°C for 24 h. The isolated strains were stained using Gram staining, and the physicochemical indexes of the isolated strains were determined through micro biochemical identification based on the manual for the identification of common bacterial systems [6].

### 16S ribosomal RNA sequencing analysis of the isolates

The isolated strains were inoculated into agar medium plates with brain heart extract and cultured at 28 °C for 18 h. A single colony was selected and placed in 10 μl of sterile water, and used as a template for PCR.

The universal primers used for 16S rRNA sequencing were 27F: 5’-agagtttgatc (c/a) tggctcag-3’, and 1492R: 5’-ggttaccttgttacgatt-3’ [7]. PCR reaction system: 50 μl of 2 × Taq PCR mix, 47 μl of ddH_2_O, 1 μl of upstream and downstream primers, and 1 μl of the template. Reaction conditions: denaturation at 95 °C for 1 min, 35 cycles of equilibrium at 98 °C for 15 s, annealing at 55 °C for 30 s and extension at 72 °C for 2 min, followed by incubation at 72 °C for 10 min. The amplified product was verified by 1% agarose gel electrophoresis to match the target fragment size and then sent to Shanghai Bioengineering for purification and sequencing. The 16S rRNA gene sequence of the NW01 strain was added to NCBI for comparison. The 16S rRNA sequences of *Vibrio* and important aquatic pathogens were selected and analysed using Cluster x software. The phylogenetic tree was constructed using the neighbour joining method in MEGA 6.0 software and the confidence interval of the bootstrapping was 10 000 times.

### Pathogenicity

According to Koch's rule, the regression infection experiment was designed to determine whether the isolate was pathogenic to bullfrogs and to confirm whether the isolate was the pathogen that caused disease in bullfrogs. The isolated strains were cultured in brain heart extract medium at 28 °C for 18 h. The bacteria were collected at 4000 rpm for 5 min at 4 °C, and then the bacterial mass was resuspended in sterile PBS buffer. The concentration of the bacterial suspension was adjusted to 10^4^, 10^6^ and 10^8^ CFU/ml. One hundred and twenty healthy bullfrogs were randomly divided into four groups (A, B, C and D) with 30 frogs in each group. Groups A, B and C were used as the experimental groups, while group D was used as the control group. The bullfrogs in groups A–C were intraperitoneally injected with 0.1 ml of the bacterial suspension, and the concentrations of the bacterial suspensions used were 10^8^, 10^6^ and 10^4^ CFU/ml respectively, the injection doses were 10^7^, 10^5^ and 10^3^ CFU/frog, while the bullfrogs in group D were injected with the same dose of PBS at the same site. During the experiment, the air temperature was kept constant at 24–26 °C and ventilation was also kept constant. Fully aerated tap water was used for breeding. The amount of water used for breeding did not exceed the neck of the bullfrogs. Each bucket was covered with a grey cover to prevent the frog from escaping. The water used for breeding was changed every day. The state of the experimental bullfrogs was observed until death. Clinical symptoms and mortality were recorded every day, and bacteria were isolated from the dying bullfrog and purified. The purified bacteria were identified using 16S rRNA sequencing.

### Analysis of drug sensitivity of the NW01 strain

A standard NCCLS antimicrobial susceptibility test was conducted using the paper diffusion method to analyse the antimicrobial susceptibility of the isolates [8]. The NW01 isolate was inoculated into a nutrient broth and cultured at 28 °C for 24 h at 200 rpm. The bacterial suspension was diluted with PBS to a concentration of 10^7^ CFU/ml. In total, 100 μl of the bacteria suspension was used to coat MH agar gel, and the selected drug-sensitive paper was pasted on the plate. The drug content of the paper is shown in the table. The plate was incubated at 28 °C for 24 h, and the size of the inhibition zone was measured.

### Serotype identification of the isolate

The serotype of the isolated bacteria was identified based on the method used for the *Vibrio cholerae* O antigen diagnostic serum. First, the suspension of bacteria to be evaluated was dripped onto a clean slide, and then a single drop of *V. cholera* O1 group, O1 group Inaba type, O1 group Ogawa type, O139 group diagnostic serum was dripped onto the suspension of bacteria to be tested, and mixed evenly, and observed 1 min later. Meanwhile, physiological saline was used as the control. Agglutination at 2+ or more was considered as a positive result.

## Results

### The epidemic time and clinical symptoms of the diseased bullfrog

**Fig. 1.**
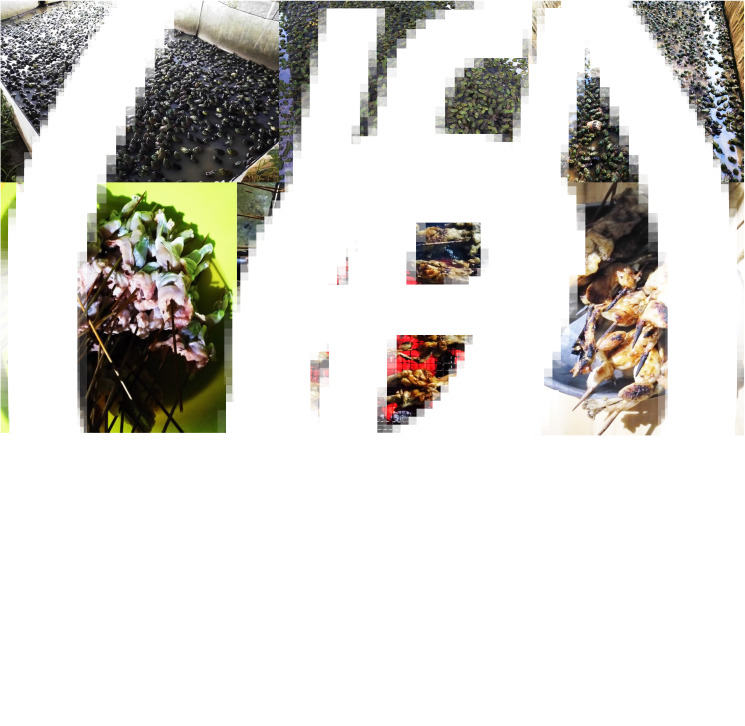
Cultivation and feed for bullfrog: (a)–(c) cultivation; (d, e) feed.

**Fig. 2.**
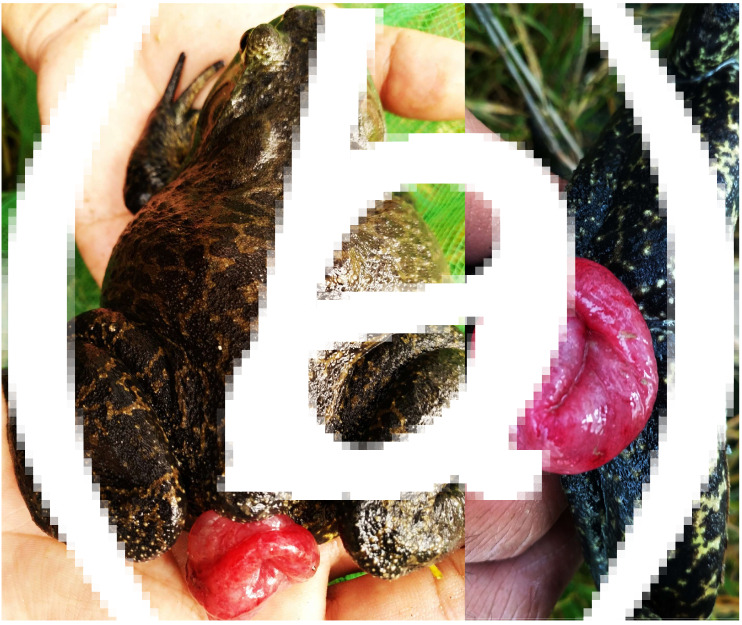
A diseased bullfrog.

Epidemiological investigations showed that the disease affected the entire breeding cycle of the bullfrogs, especially during the warm season. The increase in feeding intensity (Fig. 1a–c) made the intestinal tract of bullfrogs more prone to inflammation, leading to anal abscission (Fig. 2). The diseased bullfrogs showed no obvious symptoms on the surface of their body. Dissection showed that the diseased bullfrogs suffered slight congestion and swelling of the viscera, severe intestinal inflammation and rotten faeces. Based on these symptoms, the disease was named as bullfrog enteritis. The weight of the diseased bullfrogs ranged from 100 to 1000 g, and there were no significant individual differences. During the investigation, the incidence rate of enteritis in bullfrogs was high, but mortality rate was not high, indicating the prevalence of a chronic disease. Enteritis can lead to indigestion, malnutrition and intestinal mucosa damage of the bullfrog, which makes it easier for other pathogens, such as *Streptococcus* infection, to occur. In addition, enteritis can cause anal prolapse in bullfrogs, which seriously affects the appearance of commercially available bullfrogs, resulting in exceptionally large economic losses for farmers. No parasitic or fungal infections were found in bullfrogs when observed under a light microscope. After the bacteriological study, a strain of bacteria was isolated, purified and was temporarily named as NW01.

### Biochemical characterisation and molecular identification of the bacteria

**Fig. 3.**
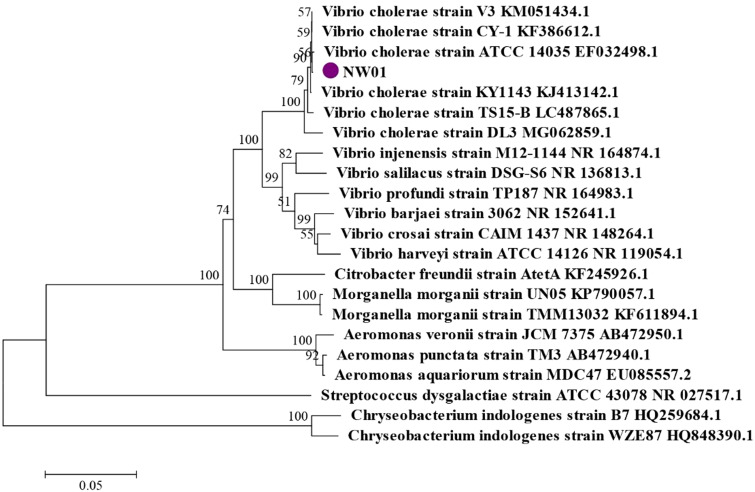
The phylogenetic tree constructed bases on the 16s rRNA sequence o.

The results of Gram staining showed that the NW01 isolate was red, indicating that it was a Gram-negative bacterium. The specific physical and chemical characteristics of the bacteria are shown in Table 1. The physical and chemical characteristics identified NW01 as *V. cholerae*. The 16S rRNA gene of NW01 was amplified using universal primers, and the 16S rRNA fragment of NW01 was about 1500 bp long, which is in line with the expected size. The 16S rRNA gene sequence (GenBank accession number: MT126343) of the isolated strains was added to a gene library and analysed using the NCBI-BLAST program. The result showed that the isolate showed the highest degree of homology with *V. cholerae*. The 16S rRNA gene sequences of several *Vibrio* species and important aquatic pathogens were selected to construct a phylogenetic tree, as shown in Figure 3. The results showed that the isolate and *V. cholerae* were clustered into one branch. Therefore, the combination of physical and chemical characteristics and gene analysis of the isolate confirmed the identity of the NW01 isolate as *V. cholerae*.

**Table 1.** Physiological and biochemical characteristics of the NW01 strain

Characteristic	*V. cholerae*	NW01
Arginine dihydrolase	–	–
V – p reaction	+	+
Oxidase	+	+
Nitrate	+	+
D glucose gas production	–	–
Amylase	+	+
Gelatinase	+	+
Lipase	+	+
Chitinase	+	+
L-leucine	–	–
L-malate	+	+
Moveability	–	–
D-mannose	+	+
Propyl alcohol	–	–
Pyruvic acid	+	+
L-rhamnose	–	–
Salicin	–	–
D-ribose	+	+
D-sorbitol	–	–
D-xylose	–	–
Trehalose	+	+
L-tyrosine	–	–
Melibiose	–	–
L-ornithine	+	+
L-proline	+	+

+, positive; –, negative.

### Pathogenicity

**Fig. 4.**
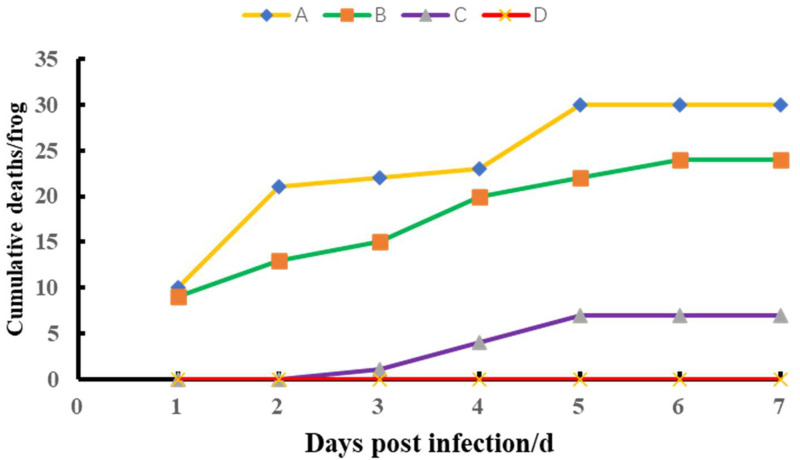
The pathogenicity of healthy bullfrogs experimentally infected with 10^7^ (a), 10^5^ (b), 10^3^ (c) CFU/bullfrog doses of the isolated NW01 strain or 0.1 ml of PBS (d).

In the pathogenicity study of the isolate, each experimental group showed a different degree of mortality (Fig. 4). The mortality rates of groups A, B and C were 100%, 80% and 23.33%, respectively. The dead bullfrogs showed similar symptoms to natural disease, while bullfrogs in the control group did not show signs of disease or mortality. *Vibrio cholerae* was isolated again from the dying bullfrogs. The infection experiment was performed in accordance with Koch's law, and the results indicated that *V. cholerae* was the pathogen that had caused bullfrog enteritis.

### Drug sensitivity tests conducted on the NW01 strain

The sensitivity of NW01 to 20 antibiotics was determined. The results showed that NW01 was resistant to *β*-lactams, aminoglycosides, macrolides, tetracyclines and sulfonamides, but sensitive to cephalosporins, quinolones and amido alcohols. Among the drugs it was sensitive to neomycin, doxycycline and florfenicol are allowed to be used in aquaculture (Table 2). Therefore, neomycin can be used for a course of 7 days to control the spread of the disease, but it cannot easily improve bullfrog anal prolapse. Therefore, neomycin was selected for the treatment of bullfrog enteritis.

**Table 2.** Susceptibility of NW01 to antibiotics

Drug		The judgment standard of inhibition zone diameter (mm)			Dose (g/piece)	Inhibition zone diameter (mm)
		Resistant	Intermediately sensitive	Sensitive		
*β*-lactam	Penicillin	≤17	18–20	≥21	10U	0^R^
	Amoxicillin	≤13	14–17	≥18	20	0^R^
Cephalosporin	Cefazolin oxime	≤14	15–19	≥20	30	30 ± 0.19^S^
	Cefradine	≤14	15–17	≥18	30	25 ± 0.14^S^
	Cefotaxime	≤14	15–22	≥23	30	22 ± 0.10^I^
Aminoglycosides	Gentamicin	≤12	13–14	≥15	10	10 ± 0.21^R^
	Streptomycin	≤11	12–14	≥15	10	9 ± 0.11^R^
	Netilmicin	≤12	13–14	≥15	30	13 ± 0.17^I^
	Kanamycin	≤13	14–17	≥18	30	19 ± 0.12^S^
	Tobramycin	≤12	13–14	≥15	10	10 ± 0.18^R^
	Neomycin^a^	≤12	13–16	≥17	30	20 ± 0.22^S^
Macrolides	Azithromycin	≤13	14–17	≥18	15	16 ± 0.31^I^
	Erythromycin	≤13	14–22	≥23	15	12 ± 0.33^R^
Tetracyclines	Tetracycline	≤18	19–22	≥23	30	18 ± 0.14^R^
	Doxycycline^a^	≤12	13–15	≥16	30	22 ± 0.25^S^
Quinolones	Enoxacin	≤14	15–17	≥18	10	25 ± 0.18^S^
	Norfloxacin	≤12	13–16	≥17	10	27 ± 0.17^S^
Amphenicols	Chloramphenicol	≤12	13–17	≥18	300	30 ± 0.14^S^
	Florfenicol^a^	≤12	13–17	≥18	75	28 ± 0.19^S^
Sulfonamides	Sulfamisoxazole^a^	≤12	13–16	≥17	300	10 ± 0.16^R^

*Note*: Data are presented as mean ± standard deviation; S, sensitive; I, intermediately sensitive; R, resistant.

a Veterinary antibiotics used in aquaculture.

### Serotype identification of the isolated bacteria

The O antigen slide agglutination test was conducted, and it was found that the isolated strain NW01 did not agglutinate with the *V. cholerae* O1 group, O1 group rice leaf type, O1 group Ogawa type or O139 diagnostic serum, and the isolate was further identified as non-O1/non-O139 group *V. cholerae*.

## Discussion

China has a long history of breeding frogs. Frogs are not only bred for use as food but are also harvested to produce a variety of industrial raw materials and provide good economic benefits. Bullfrog is an important representative species [9]. Along with large developments in the bullfrog breeding industry, diseases are more frequently observed during the breeding process. Due to large-scale domestic bullfrog breeding, reports on bullfrog diseases have been mainly concentrated in China [4, 10]. Overseas, only South Korea, France, North America and a few other countries have reported of the same. The main pathogens of bullfrog diseases include viruses, bacteria and parasites [11–16]. The frequent occurrence of diseases in bullfrog has caused great economic losses, and diseases were becoming a major bottleneck for the development of the bullfrog industry.

In this study, the dominant strain NW01 was isolated from diseased bullfrogs. The isolated NW01 was identified as *V. cholerae* through biochemical identification, 16S rRNA sequence analysis and construction of a phylogenetic tree. The regression infection experiments confirmed that *V. cholerae* was the pathogen that caused bullfrog enteritis. The results showed that *V. cholerae*, a zoonotic bacterium caused the first infection in the bullfrogs, which led to a great epidemic of diseases and caused great economic losses.

Based on the sensitivity of the isolates to 20 types of antibiotics, neomycin was selected to be used for clinical prevention and control in this study, and the spread of the epidemic was controlled in time. However, it cannot induce a good therapeutic effect on bullfrog anal prolapse, a serious condition that is difficult to improve.

*Vibrio cholerae* belongs to the *Vibrio* family and can be divided into 139 serogroups. Among them, O1 and O139 can cause cholera in humans. The O1 and O139 groups cause cholera mainly because they carry the cholera toxin [17, 18], which activate adenylate cyclase in intestinal epithelial cells, resulting in the secretion of Cl^−^ ions and impairment of Na^+^ ion absorption. Water enters the intestinal cavity along with ions, causing severe watery diarrhoea, which can lead to human death. Since 1817, there have been seven cholera pandemics worldwide that have caused hundreds of millions of human deaths. However, non-O1 and non-O139 *V. cholerae* carry other virulence factors, which are widely distributed in the water environment. They can usually cause human gastrointestinal inflammation and may sometimes extraintestinal infections, such as meningitis, sepsis and wound infections [19].

*Vibrio cholerae* widely exists in all types of waterbodies [19, 20] and it has been reported that *V. cholerae* can infect aquatic animals including fish [21, 22], shrimps [23, 24] and other aquaculture animals [25, 26]. The cause of cholera epidemics is extraordinarily complex, and it is unclear how it spreads, while the reason for seasonal epidemic peaks in epidemic areas are also unknown. However, it is an indisputable fact that cholera is caused by *V. cholerae* [27].

Since the transmission mechanism of *V. cholerae* is not clear, it has been thought that aquatic animals are infected with non-O1 and non-O139 *V. cholerae*. The *V. cholerae* isolated from bullfrogs were also non-O1 group and non-O139 group, which can cause symptoms of intestinal inflammation and even anal prolapse, resulting in low levels of mortality and a long duration of survival, but a high incidence rate.

However, further research has shown that O1 and O139 group *V. cholerae* have been reported in aquatic animals, such as the reports of O1 group found in tilapia [28] and O139 group found in loach and shrimp [29, 30]. These reports indicate that *V. cholerae* in aquatic animals is not all non-O1 and non-O139 groups, but may also be O1 and O139 groups, which can cause cholera outbreaks. Therefore, *V. cholerae* is an important zoonotic bacterium that we must be mindful of.

At present, there are many methods in which bullfrogs are used as food, among which hot pot bullfrogs and barbecued bullfrogs are the two main ways, and the bullfrogs prepared using these methods may be eaten without being fully cooked (Fig. 1d and f). The results of our study have indicated that bullfrogs are likely to be infected with *V. cholerae* through aquatic products. Other studies have found *Salmonella* and microsporidia in bullfrogs, which are also serious zoonotic pathogens [3, 31]. Although only non-O1 and non-O139 *V. cholerae* were found in bullfrogs in this study, it is possible that *V. cholerae* can mutate and become O1 and O139 serotypes through gene transfer under the current open culture practice of bullfrogs [32]. Therefore, these findings need to be given appropriate attention.

## Data Availability

All data generated or used during the study appear in the submitted article.
